# Pesticide Exposure, Safety Issues, and Risk Assessment Indicators

**DOI:** 10.3390/ijerph8051402

**Published:** 2011-05-06

**Authors:** Christos A. Damalas, Ilias G. Eleftherohorinos

**Affiliations:** 1 Department of Agricultural Development, Democritus University of Thrace, Pantazidou 193, 682 00 Orestiada, Greece; 2 Laboratory of Agronomy, School of Agriculture, Aristotle University of Thessaloniki, University Campus, 541 24 Thessaloniki, Greece; E-Mail: eleftero@agro.auth.gr

**Keywords:** pesticide toxicity, pesticide safety, risk assessment

## Abstract

Pesticides are widely used in agricultural production to prevent or control pests, diseases, weeds, and other plant pathogens in an effort to reduce or eliminate yield losses and maintain high product quality. Although pesticides are developed through very strict regulation processes to function with reasonable certainty and minimal impact on human health and the environment, serious concerns have been raised about health risks resulting from occupational exposure and from residues in food and drinking water. Occupational exposure to pesticides often occurs in the case of agricultural workers in open fields and greenhouses, workers in the pesticide industry, and exterminators of house pests. Exposure of the general population to pesticides occurs primarily through eating food and drinking water contaminated with pesticide residues, whereas substantial exposure can also occur in or around the home. Regarding the adverse effects on the environment (water, soil and air contamination from leaching, runoff, and spray drift, as well as the detrimental effects on wildlife, fish, plants, and other non-target organisms), many of these effects depend on the toxicity of the pesticide, the measures taken during its application, the dosage applied, the adsorption on soil colloids, the weather conditions prevailing after application, and how long the pesticide persists in the environment. Therefore, the risk assessment of the impact of pesticides either on human health or on the environment is not an easy and particularly accurate process because of differences in the periods and levels of exposure, the types of pesticides used (regarding toxicity and persistence), and the environmental characteristics of the areas where pesticides are usually applied. Also, the number of the criteria used and the method of their implementation to assess the adverse effects of pesticides on human health could affect risk assessment and would possibly affect the characterization of the already approved pesticides and the approval of the new compounds in the near future. Thus, new tools or techniques with greater reliability than those already existing are needed to predict the potential hazards of pesticides and thus contribute to reduction of the adverse effects on human health and the environment. On the other hand, the implementation of alternative cropping systems that are less dependent on pesticides, the development of new pesticides with novel modes of action and improved safety profiles, and the improvement of the already used pesticide formulations towards safer formulations (e.g., microcapsule suspensions) could reduce the adverse effects of farming and particularly the toxic effects of pesticides. In addition, the use of appropriate and well-maintained spraying equipment along with taking all precautions that are required in all stages of pesticide handling could minimize human exposure to pesticides and their potential adverse effects on the environment.

## 1. Introduction

Pesticides are widely used in most sectors of the agricultural production to prevent or reduce losses by pests and thus can improve yield as well as quality of the produce, even in terms of cosmetic appeal, which is often important to consumers [1,2]. Pesticides can also improve the nutritional value of food and sometimes its safety [3,4]. There are also many other kinds of benefits that may be attributed to pesticides, but these benefits often go unnoticed by the general public [2,5]. Thus, from this point of view, pesticides can be considered as an economic, labor-saving, and efficient tool of pest management with great popularity in most sectors of the agricultural production.

Despite their popularity and extensive use, pesticides serious concerns about health risks arising from the exposure of farmers when mixing and applying pesticides or working in treated fields and from residues on food and in drinking water for the general population have been raised [6–10]. These activities have caused a number of accidental poisonings, and even the routine use of pesticides can pose major health risks to farmers both in the short and the long run and can degrade the environment. In developing countries, farmers face great risks of exposure due to the use of toxic chemicals that are banned or restricted in other countries, incorrect application techniques, poorly maintained or totally inappropriate spraying equipment, inadequate storage practices, and often the reuse of old pesticide containers for food and water storage [11–13]. Obviously, exposure to pesticides poses a continuous health hazard, especially in the agricultural working environment. By their very nature most pesticides show a high degree of toxicity because they are designed to kill certain organisms and thus create some risk of harm. Within this context, pesticide use has raised serious concerns not only of potential effects on human health, but also about impacts on wildlife and sensitive ecosystems [14–16]. Often, pesticide applications prove counterproductive because they kill beneficial species such as natural enemies of pests and increase the chances of development of pest resistance to pesticides. Furthermore, many end users have poor knowledge of the risks associated to the use of pesticides, including the essential role of the correct application and the necessary precautions [17–20]. Even farmers who are well aware of the harmful effects of pesticides are sometimes unable to translate this awareness into their practices [21–24].

Although pesticides have been developed to function with reasonable certainty and minimal risk to human health and the environment, the published results are not always in agreement with this fact. Even though the development of toxicity reference levels for pesticides incorporates uncertainty factors that serve to achieve this regulatory standard, in reality, we may never know whether a pesticide is safe under all circumstances, nor can we predict with certainty its performance in hypothetical situations. Scientific investigation is bound by the tools and the techniques that are available and therefore new developments continually redefine our capabilities. Despite many studies on the fate and toxicity of pesticides, there are research gaps causing uncertainty in the predictions of their long-term health and environmental effects. On the basis of these contradictory results of the literature, discussions among scientists and the public focused on the real, predicted, and perceived risks that pesticides pose to human health (worker exposure during pesticide use and consumer exposure to pesticide residues found in fresh fruit, vegetables and drinking water) and the environment (water and air contamination, toxic effects on non-target organisms) are fully justified [5,8,25,26].

The purpose of this paper is to present and discuss: (1) basic safety issues related to pesticide registration, (2) common factors affecting exposure to pesticides, and (3) common indicators used for the prediction of the adverse effects of pesticides on human health and the environment as well as their reliability and accuracy in the risk assessment of those adverse effects. It is worth mentioning that this paper does not focus on the fate of pesticides in the environment or their adverse effects on specific non-target organisms.

## 2. Pesticide Registration and Safety

Pesticide registration is a scientifically-based, legal, and also administrative process, where a wide variety of effects associated with the use of a pesticide product and its potential effect on human health and the environment is assessed [27–29]. The registration is an important step in the management of pesticides as it enables authorities primarily to determine which pesticide products are permitted to be used and for what purposes, and also to exercise control over quality, usage rates, claims, labelling, packaging and advertising of pesticides, thus ensuring that the best interest of end-users as well as the environment are well protected [30]. In addition, the registration process is restricted to the assumption that pesticides are only used for their intended function and envisages proving that such use does not promote unreasonable effects either on human health or on the environment. Therefore, before any pesticide can be used commercially, several tests are conducted that determine whether a pesticide has any potential to cause adverse effects on humans and wildlife, including endangered species and other non-target organisms, or potential to contaminate surface waters and groundwater from leaching, runoff, and spray drift. Effects in any non-target species may translate into ecosystem unbalance and food-web disruption that ultimately may affect human health and edible species.

Pesticide registration is a complex process and takes considerable time, resources, and expertise on the part of the registration authority, the pesticide manufacturing industry, and various public interest groups. An expanding series of tests based on improved technology is used to provide precise pesticide residue detections and toxicological assessments in response to public concern. In addition, improved methods for hazard predictions, novel approaches to hazard reduction measures, and incorporation of the broadening scope of relevant scientific knowledge into industry and government policy decisions contribute to changes and improvements in the pesticide registration process.

The basic pathway for the registration of a pesticide is: (1) research conducted by the manufacturer prior to its decision to pursue registration; (2) submission of data report by the manufacturer to the registration authority; (3) review of the data by the registration authority; and (4) a decision by the registration authority either to register the pesticide, based on the merits of the submitted data, or to deny registration. The decisions of the registration authority to register a pesticide hinges on a benefit-to-risk analysis of the required data. Therefore, it is essential that all steps in the registration process are transparent, based on sound and published criteria and guidance documents, with full information shared with the applicant on the outcomes of the various steps in the registration procedure [31]. Also, the registration authority ensures that each registered pesticide continues to meet the highest standards of safety to protect human health and the environment as these standards are becoming stricter over the years with regard to our ability to evaluate the potential effects of pesticides. Within this context, older pesticides are being reviewed to ensure that they meet current scientific and regulatory standards. This process, called re-registration, considers the human health and ecological effects of pesticides and results in actions to reduce risks that are of concern. Indeed, very drastic changes have occurred in the list of legally marketed pesticides over the last years in the EU as a result of the EU legislation on marketed pesticides, which was enacted in 1993 (with Directive 91/414/EEC) and lasted effectively until December 2008. During this period, approximately 704 active substances were banned, of which 26% were insecticides, 23% herbicides and 17% fungicides [32]. Also, EPA in USA has completed several individual pesticide re-registration and tolerance reassessment decisions (the results of reviews are summarized in Re-registration Eligibility Decision documents), which improved food safety, human health and environmental protection in the United States [29].

The registration process for a pesticide usually requires the manufacturer (registrant) to conduct, analyze, and pay for many different scientific tests. These tests define the product chemistry, risks to humans and domestic animals, the environmental fate of the pesticide, and the impact on non-target organisms [30,31]. Data required to support an application of a registration should cover all relevant aspects of the product during its full life-cycle. They should include the identity and physical and chemical properties of the active ingredient and formulated product, analytical methods, human and environmental toxicity, proposed label and uses, safety data sheets, efficacy for the intended use as well as residues resulting from the use of the pesticide product, container management, and waste product disposal. Generation of such data for a single compound may take several years and costs a great amount of money. Also, toxicological testing is conducted under stringent guidelines, approved methodologies, and specified reporting requirements. Exacting standards are necessary for consistency in the evaluations of pesticide safety and also for the comparisons among chemicals. Ecological risk assessments to determine what risks are posed by a pesticide and whether changes to the proposed use(s) of the product are necessary to protect human health, wildlife, and the environment. To evaluate the environmental risks of a pesticide product, scientists of the registration authority look at all the data together. If the risk assessment indicates a high likelihood of hazard to wildlife or any phytotoxicity to non-target plants, the registration authority may require additional testing and extra data or require that the pesticide be applied only by certified individuals (*i.e.*, restricted use). Alternatively, the registration authority may decide not to allow its use.

## 3. Human Exposure to Pesticides and Factors Affecting Exposure

Human exposure to pesticides may occur through occupational exposure in the case of agricultural workers in open fields and greenhouses, workers in the pesticide industry, and exterminators of house pests [6–10,33–35]. However, irrespective of whether the occupation involves the use of pesticides, the presence of such chemicals in the working environment constitutes potential occupational exposure. Evidently, workers who mix, load, transport and apply formulated pesticides are normally considered to be the group that will receive the greatest exposure because of the nature of their work and are therefore at highest risk for possible acute intoxications [36]. In some situations, exposure to pesticides can occur from accidental spills of chemicals, leakages, or faulty spraying equipment. The exposure of workers increases in the case of not paying attention to the instructions on how to use the pesticides and particularly when they ignore basic safety guidelines on the use of personal protective equipment and fundamental sanitation practices such as washing hands after pesticide handling or before eating.

Several factors can affect exposure during pesticide handling [36]. The form of formulation of pesticide products may affect the extent of exposure. Liquids are prone to splashing and occasionally spillage, resulting in direct skin contact or indirect skin contact through clothing contamination. Solids may generate dust while being loaded into the application equipment, resulting in exposure to the face and the eyes and also respiratory hazards. The type of packaging of pesticide products can also affect potential exposure. For example, the opening of pesticide bags can result in some kind of exposure depending on the type of packaging in combination with the formulation of the active ingredient. Also, the size of cans, bottles, or other liquid containers may affect the potential for spillage and splashing. Moreover, adjuvant chemicals used in pesticide formulations to enhance their efficiency in terms of biological activity (e.g., enhance the contact between the active ingredient and its specific molecular target) as well as to facilitate application and reaching target species, may show toxicity themselves, thus contributing to the overall effect of exposure to a commercial pesticide product [37]. Weather conditions at the time of application, such as air temperature and humidity, may affect the chemical volatility of the product, the perspiration rate of the human body, and the use of personal protective equipment by the users [36,38–40]. Wind increases considerably spray drift and resultant exposure to the applicator. The amount of pesticide that is lost from the target area and the distance the pesticide moves will increase as wind velocity increases, so greater wind speed generally will cause more drift. In addition, low relative humidity and high temperature will cause more rapid evaporation of spray droplets between the spray nozzle and the target than high relative humidity and low temperature. General hygiene behaviour of workers during pesticide use can also have substantial impact on exposure. For example, workers who avoid mixing and spraying during windy conditions can reduce the exposure. Proper use and maintenance of protective clothing are considered important behaviours associated with reduced chemical exposures. Furthermore, the frequency and duration of pesticide handling both on a seasonal and lifetime basis affects the exposure. In particular, the exposure of an individual farmer that applies a pesticide once a year is lower than that of a commercial applicator that normally applies a pesticide for many consecutive days or weeks in a season [36].

Exposure of the general population to pesticides occurs mainly through eating food and drinking water contaminated with pesticides, whereas substantial exposure to pesticides can also occur when living close to a workplace that uses pesticides or even when workers bring home contaminated articles [41,42]. Non-occupational exposure originating from pesticide residues in food, air and drinking water generally involves low doses and is chronic (or semi-chronic). However, clear links between individual pesticides and individual health effects can only be shown in animal studies, but the doses used in these studies are far higher than the enforced legally pesticide limits [43]. Therefore, the risk to human health from these studies appears to be negligible. The actual acute exposure, however, may be higher than that anticipated due to certain food preferences, residue variability between individual food items and the greater than average consumption of a particular food item only at one sitting [44]. As a result of pesticide use in or around the home, individuals can be exposed during the preparation and application of pesticides or even after the applications are completed, whereas delayed exposure can occur through inhalation of residual air concentrations or exposure to residues found on surfaces, clothing, bedding, food, dust, discarded pesticide containers, or application equipment [41]. Also, accidental poisoning with pesticides in the home is a possibility from pesticide use around the house or garden. Exposure is likely to occur from pesticide spills, improper use, or poor storage as a result of use without reading or accounting to the pesticide label. Pesticide mishandling such as transferring the products from their original packages into household containers and also the lack of compliance with instructions of the label can be also sources of exposure [42].

## 4. Pesticide and Human Health

Risk assessment of pesticide impact on human health is not an easy and particularly accurate process because of differences in the periods and the levels of exposure, type of pesticides (regarding toxicity), mixtures or cocktails used in the field, and the geographic and meteorological characteristics of the agricultural areas where pesticides are applied [45,46]. Such differences refer mainly to the people who prepare the mixtures in the field, the pesticide sprayers, and also the population that lives near the sprayed areas, pesticide storage facilities, greenhouses, or open fields. Therefore, considering that human health risk is a function of pesticide toxicity and exposure, a greater risk is expected to arise from high exposure to a moderately toxic pesticide than from little exposure to a highly toxic pesticide. However, whether or not dietary exposure of the general population to pesticide residues found on food and drinking water consists of a potential threat to human health, is still the subject of great scientific controversy [47].

Regardless of the difficulties in assessing risks of pesticide use on human health, the authorization for pesticide commercialization in Europe currently requires data of potential negative effects of the active substances on human health. These data are usually obtained from several tests focused on e.g., metabolism patterns, acute toxicity, sub-chronic or sub-acute toxicity, chronic toxicity, carcinogenicity, genotoxicity, teratogenicity, generation study, and also irritancy trials using rat as a model mammal or in some cases dogs and rabbits [48]. The respective toxicity tests for human health risk assessments required by EPA [29] are (1) the acute toxicity test, which assesses the effects of short-term exposure to a single dose of pesticide (oral, dermal, and inhalation exposure, eye irritation, skin irritation, skin sensitization, neurotoxicity), (2) the sub-chronic toxicity test, which assesses the effects of intermediate repeated exposure (oral, dermal, inhalation, nerve system damage) over a longer period of time (30–90 days), (3) the chronic toxicity test, which assesses the effects of long-term repeated exposure lasting for most of the test animal’s life span and intended to determine the effects of a pesticide product after prolonged and repeated exposures (e.g., chronic non-cancer and cancer effects), (4) the developmental and reproductive tests, which assess any potential effects in the fetus of an exposed pregnant female (*i.e.*, birth defects) and how pesticide exposure may influence the ability of a test animal to reproduce successfully, (5) the mutagenicity test which assesses the potential of a pesticide to affect the genetic components of the cell, and (6) the hormone disruption test, which measures the pesticide potential to disrupt the endocrine system (consists of a set of glands and the hormones they produce that regulate the development, growth, reproduction, and behavior of animals including humans). The acute toxicity experiments are required for the calculation of the median lethal dose (LD_50_), which is the pesticide dose that is required to kill half of the tested animals when entering the body by a particular route. For example, if the substance is swallowed the figure is an oral LD_50_, whereas if absorbed through the skin it is a dermal LD_50_. In addition, the acute inhalation lethal concentration (LC_50_), which is the pesticide concentration required to kill half of the exposed (for 4 hours) tested animals to a pesticide, is also calculated. Lethal concentration values are used when the route of administration is by inhalation or intake via drinking water (rather than oral, dermal, *etc.*). These endpoints are used for WHO and EPA toxicity classifications of pesticides shown in Tables 1, 2, and 3.

The oral LD_50_ is usually lower than the dermal LD_50_ since pesticides can enter the bloodstream more easily through the stomach than through the skin [49]. It must be noted that the LD_50_ values given in the WHO classification are for the active ingredient, whereas these LD_50_ values must be modified to take account of the concentration of the pesticide formulation actually used. This is because the actual toxicity of a commercial pesticide product is significantly affected by the formulation. For example, a highly toxic pesticide becomes more toxic when is formulated as emulsifiable concentrate than as microcapsule suspension [50]. This is because the amount of the toxic active ingredient at the time of application from the emulsifiable concentrate is much higher than that of the microcapsule suspension. In addition, the emulsifiable concentrate is more toxic than the microcapsule suspension because it includes very often toxic organic solvents [37]. Also, the toxicity of the liquid formulation is usually much higher than that of the respective solid formulation since it is more difficult for a solid to pass through the skin [51].

Long-term studies exposing test animals at a range of pesticide doses allow defining the reference point below of which no adverse effects occur. This dose (reference point), known as No Observed Adverse Effect Level (NOAEL) or No Observed Effect Level (NOEL), is used to derive the acceptable daily intake (ADI) for humans, which is defined as the amount of chemical that can be consumed every day for a lifetime with no harm. It is worth mentioning that a 100-fold safety or uncertainty factor is taken into account in calculating the safe daily intakes of food by humans. This is done to overcome differences between animals that are used in the tests as well as differences between humans (inter-individual variability).

The Acute Reference Dose (ARfD) is also calculated for cases that people intake much higher levels of a pesticide than the ADI as a result of consuming certain food items (with differential pesticide contamination of the different food items) only at once. The value of ARfD is based on the lowest NOAEL, but is adjusted by an appropriate uncertainty factor. For individuals who work with pesticides regularly, the Acceptable Operator Exposure Level (AOEL) is calculated on the basis of short-term toxicity studies related to the oral route of pesticides [48].

Pesticides are additionally classified according to the principles of the International Agency for Research on Cancer (IARC) [52] (often cited as IARC class). The classification of a pesticide in this category reflects the strength of the evidence derived from epidemiological studies in humans, from experiments with animals, and from mechanistic and other relevant data. A pesticide is classified in this category when there is sufficient evidence of carcinogenicity in humans. Exceptionally, a pesticide may be placed in this category when evidence of carcinogenicity in humans is less than sufficient, but there is sufficient evidence of carcinogenicity in experimental animals and strong evidence in exposed humans that the pesticide acts through a relevant mechanism of carcinogenicity. According to IARC classification, a pesticide is classified in group 1, if it is carcinogenic to humans; in group 2A, if it is probably carcinogenic to humans (when there is limited evidence of carcinogenicity in humans and sufficient evidence of carcinogenicity in test animals); in group 2B, if it is possibly carcinogenic to humans (e.g., limited evidence of carcinogenicity in humans and less than sufficient evidence in test animals); in group 3, if it is not classifiable as to its carcinogenicity to humans (inadequate evidence of carcinogenicity in humans and inadequate or limited evidence in experimental animals); and in group 4, if it is probably not carcinogenic to humans. The respective carcinogenicity classes of EPA are as follows: (1) carcinogenic to humans, (2) likely to be carcinogenic to humans, (3) suggestive evidence of carcinogenic potential, (4) inadequate information to assess carcinogenic potential, and (5) not likely to be carcinogenic to humans [29].

The results on toxicity characterization (based on the databases of EPA, IARC, WHO, and Pesticide Action Network) of the 276 legally marketed active substances in Europe indicate that 32 out of the 76 fungicides, 25 out of the 87 herbicides and 24 out of the 66 insecticides are related to at least one health effect (e.g., carcinogenic, endocrine disruptor, reproductive and developmental toxicity, acute toxicity) [32]. In particular, 51 and eight pesticides (including fungicides, herbicides, and insecticides) are characterized as carcinogenic according to EPA and IARC databases, respectively, 24 pesticides are characterized as endocrine disruptors (based on the database of the Pesticide Action Network), 22 pesticides are characterized as presenting reproductive and developmental toxicity (Pesticide Action Network), and 28 pesticides as presenting acute toxicity (based on WHO classification).

Eighty-four out of the 276 approved active substances (81 of them are pesticides) in Europe were characterized as toxic (have at least one adverse health effect characterization) by Karabelas *et al.* [44]. However, different results on the number of toxic pesticides were reported by KEMI [53] for Swedish Chemical Agency and by the Pesticides Safety Directorate [54] for UK. In particular, KEMI [53], taking into account the new hard cut-off criteria of the European Union (EU) for approval of active substances, found only 23 active substances (eight herbicides, 11 fungicides, three insecticides and one plant growth regulator) out of the 271 active substances (included in Annex I of 91/414/EEC Directive as well as a number of substances with decision pending) to meet the cut-off criteria of the EU and therefore would be removed. Seven of these 23 active substances have been identified as carcinogenic, mutagenic, and toxic to reproduction, 11 have been classified as endocrine disruptors, and four have been identified as persistent, bio-accumulating and toxic pollutants. The Pesticides Safety Directorate [53], considering the approval criteria adopted by the Commission’s proposal as well as in the European Parliament’s Environment, Public Health and Food Safety Committee’s, found that 60 of the 278 active substances assessed were toxic. It is worth mentioning that only 14 and 37 characterized as toxic substances by Karabelas *et al.* [32] are classified as toxic in the respective studies conducted by KEMI [53] and Pesticides Safety Directorate [54]. These results show clearly that the number of the criteria used and the method of their implementation to assess the adverse effects of pesticides on human health lead to different characterization of the already approved pesticides in Europe and would possibly affect the approval of the new compounds that will be developed in the near future.

The above findings should be interpreted with extra caution by the decision policy makers because they did not result from cause-control studies on humans, but mainly from toxicological studies on experimental animals (rats, dogs, and rabbits) and in some cases from epidemiological studies (health effects due to rather long-time human exposure to low concentrations of pesticides) associated with high uncertainty in the estimation of the relevant human exposure pattern. The fact that a very large number (∼704) of the most toxic active substances have been withdrawn in Europe over the past nine years implies that the results of epidemiological studies (where the currently banned toxic active substances unavoidably influenced the outcome) should be interpreted with extra caution as well, especially for conclusions about the present day pesticide health impact [32]. In addition, concerns by several independent scientists in Europe about the negative effects of the fewer approved pesticides should be taken into account by the policy makers on pesticide use.

## 5. Pesticide and the Environment

Pesticides, in addition to their potential negative effects on human health, pose adverse effects also on the environment (water, soil and air contamination, toxic effects on non-target organisms) [25,26]. In particular, inappropriate use of pesticides has been linked with: (1) adverse effects on non-target organisms (e.g., reduction of beneficial species populations), (2) water contamination from mobile pesticides or from pesticide drift, (3) air pollution from volatile pesticides, (4) injury on non-target plants from herbicide drift, (5) injury to rotational crops from herbicide residues remained in the field, (6) crop injury due to high application rates, wrong application timing or unfavourable environmental conditions at and after pesticide application [55].

Many of the adverse effects of pesticides on the environment depend on the interactions between the physicochemical properties (vapour pressure, stability, solubility, pK_a_) of the pesticide, soil adsorption and soil persistence, the soil factors (pH, organic components, inorganic surfaces, soil moisture, soil microflora, soil fauna), the plant species, and the climatic variation [55]. Also, the toxicity, the dosage applied, the weather conditions prevailing after the pesticide application, and how long the pesticide persists in the environment could account for its adverse effects on the environment. Soil factors and weather conditions have long been recognised as the most important factors that affect the fate of the pesticide in the environment and consequently the activity, selectivity, and adverse effects on the environment [27]. Unfortunately, since these factors vary from site to site and from year to year, the results from any field study on the fate and behaviour of the pesticide are specific for one particular location and season. Therefore, for the environmental risk assessment, the behaviour and the fate of a pesticide are initially assessed by the calculation of the predicted environmental concentration (PEC), which in the United States is referred to as estimated environmental concentration (EEC) [48]. These concentrations are calculated for soil, water, sediment, and air, and the validation is performed by comparison with the data obtained from the three levels of tests (needed for approval-registration purposes) to assess the pesticide toxicity on key non-target organisms (Table 4). Also, the toxicity exposure ratio (TER) is also calculated to determine whether the risk to the organism is acceptable or not [56]. TER is calculated from the LC_50_ or equivalent measure (LD_50_, NOEC = no observed effect concentration) of the susceptibility of an organism divided by the PEC relevant to the situation in which the organism is living. In general, a detailed higher tier risk assessment (2,3) is needed when TER is below 100, whereas a chronic risk assessment is required in the case of TER < 10. If TER is less than 5, the Annex VI of the EU Directive 91/414 EEC requires that ‘no authorisation shall be granted…unless it is clearly established through an appropriate risk assessment that under field conditions no unacceptable impact occurs after the use of the product under the proposed conditions of use’. In USA, the risk quotient (predicted exposure concentration to predicted no effect concentration) is the inverse of TER and that is calculated by dividing the PEC with the indicated toxic dose [48].

Although the agricultural soil is the primary recipient of pesticides, water bodies that are adjacent to agricultural areas are usually the ultimate recipient for pesticide residues [57]. This issue is the reason for European authorities to require data (before the pesticide commercialization in Europe) related with the risk of non-target terrestrial and aquatic organisms when addressing potential adverse effects of pesticides on the environment.

Considering the adverse effects linked with the use of pesticides in agriculture, the use of criteria to select pesticides that are effective, cost efficient and safe for the operator and the environment now appears as an imperative need [56,58,59]. Moreover, the use of certain environmental risk indicators as alternatives to direct pesticide impact measurement linked to methodological difficulties (*i.e.*, impossibility of measurement due to complexity of the system) or due to practical reasons (*i.e.*, time and costs) has also been a reality [59]. These indicators have already been used by Reus *et al*. [58] and Bockstaller *et al*. [59] to assess potential risks of pesticides for water contamination, soil organisms (mainly earthworms), bees, air emissions, bioaccumulation, and human health. Calculation of the environmental indicators used in these two studies was based on the pesticide persistence in soil (half-life, DT_50_), mobility in soil (organic-carbon adsorption coefficient, K_oc_) and toxicity to water (lethal concentration for aquatic organisms, LC_50_) and soil organisms (NOEC). Regarding the contribution of the environmental indicators on pesticide selection, the study conducted by Reus *et al*. [58] to evaluate 15 individual pesticide applications by using eight indicators showed the following: (1) some of the 15 pesticide applications had a high ranking (higher impact on the environment) with all the indicators used, but their ranking differed considerably when the score for the environment was concerned as a whole; (2) the ranking based on the indicator ‘kilograms of active ingredient’ did not correlate with most of the rankings obtained by the other pesticide risk indicators; (3) the pesticide risk indicators used gave similar rankings of the 15 pesticide applications for the individual region surface water, groundwater, and soil contamination. For the latter, the scores for surface water contamination were largely determined by the pesticide toxicity to aquatic organisms, whereas the scores for groundwater contamination were largely determined by DT_50_ and K_oc_. However, an exception was recorded with two pesticides that were found toxic or mobile although they had been applied at extreme low rates. These results indicate that new indicators with greater reliability than those already existing are needed to predict potential risk of pesticides and thus contribute to reduction of the adverse effects of pesticides on the environment [58].

## 6. Minimizing the Negative Impact of Pesticides

Despite continuing disagreements over the degree of risk posed by pesticides, it appears that people have become increasingly concerned about pesticide use and particularly about their impacts on human health and environmental quality [5]. These increased concerns resulted mainly from reduced trust in the agricultural and industrial methods of production as well as on the authority’s regulations aimed at protecting both the environment and human health. Therefore, considering the existence of several uncertainties in the evaluation of pesticide safety, scientific data, policy guidelines, and professional judgment must be incorporated when estimating whether a pesticide can be used beneficially within the limits of an acceptable risk.

The probability of reducing the environmental risk associated with the pesticide use is very low because the producers believe that lowering risk implies either decreased output or increased input resulting by the substitution for the pesticide inputs [60]. Thus, policies aiming at reducing the risks associated with the use of pesticides will impose costs on the agricultural community, which in turn has implications for agricultural commodity prices. This has been confirmed by the cost-function-based production model used by Paul *et al*. [60], which indicated that substantive costs would be imposed on the agricultural sector by the requirements to reduce environmental risk deriving from pesticide use. These costs are directly associated with increases in demand of effective pesticides, for a given level of agricultural output, and implies induced innovation to augment pesticide quality associated with increased cost.

Concerns about impacts of pesticide use on human health and the environment led the EU to develop a ‘Thematic Strategy on Sustainable Use of Pesticides’ [61]. Moreover, agricultural scientists started to develop alternative crop management systems to minimize the negative effects of farming (based mainly on pesticide use for crop protection) to the environment and to human health. In particular, the Integrated Crop Management (ICM) includes guidelines to be used by the farmer unions to enforce actions for production of safe agricultural products with simultaneous respect to the environment [25,62–65]. In addition, ICM includes measures for implementation of good agricultural practices (GAP), the safety and hygiene of workers, the safety of the products, the full traceability of the measurements, and specific actions for the preservation of the environment [66]. For the control of pests, ICM encourages the use of complementary methods of pest management (such as crop resistance against insects and fungi, biological control, and other cultural or physical measures) to reduce the animal pest or weed population below its economic injury level and to minimise pesticide impacts on other components of the agro-ecosystem [67,68]. Concerning pesticide use, ICM allows pesticide use only through an Integrated Pest Management (IPM) program [26,65,66], where certain criteria are used for pesticides selection, specific instructions are followed for their application on crops, and residue analysis is used as one of the tools for enforcement. Pesticides that are selected for use in IPM are: (1) biologically effective (high selectivity, fast impact, optimal residual effect, good plant tolerance, low risk of resistance), (2) user friendly (low acute toxicity and low chronic toxicity, optimum formulation, safe packaging, easy application method, long store stability), (3) environmentally friendly/compatible (low toxicity to non-target organisms, fast degradation in the environment, low mobility in the soil, no residues in food and fodder above the MRLs, low application rate), (4) economically viable/profitable (good cost/profit ratio for the farmer, broad spectrum of activity, applicable in IPM, innovative product characteristics, competitive, patentable) [69]. Specific instructions that are followed during pesticide application on crops include (1) the use of pesticide at the recommended dose when a pest is found or a precautionary treatment thought necessary, (2) the optimisation of pesticide use for economic saving through adjusted doses according to pest population density, and (3) the minimization of pesticide need by altering the cultivation system to lower the risk of pests [25]. Regarding the analysis of the amount of active ingredient applied or the money spent on pesticides, these variables should be used only as a first approximation, because the dosage of active ingredients is not closely related to environmental activity, while environmental friendly and innovative compounds are often more expensive than obsolete, hazardous ones. All the previously mentioned indicate clearly that the introduction of IPM system would contribute to a significant reduction of the pesticide impact on human health and the environment without affecting crop productivity or increasing the probability of crop losses [25,26,65].

Apart from the already mentioned above, chemical crop protection has been changed tremendously over the last years, not only in the development of new active ingredients, but also in the assessment of the behaviour of these chemicals in the environment, the residues in crop plants, and of their potential toxicity to humans and the environment [70–72]. This is attributed to the great scientific progress in many disciplines such as chemistry, biology, and molecular biology which has improved considerably the way of searching for new agrochemicals and the re-assessment of safety for the already used pesticides. Thus, new agrochemicals with novel modes of action and improved safety profiles are now a reality [73]. Moreover, these new agrochemicals in combination with the appropriate measures taken for safer and more effective pesticide application make the chemical crop protection as one of the most well-established technologies in agriculture which seems that it will continue to play an important role in the agribusiness in spite of the rapid emergence of novel biotechnological solutions [70,74].

## 7. Conclusions

Pesticides have played a key role in providing reliable supplies of agricultural produce at prices affordable to consumers, improving the quality of produce, and ensuring high profits to farmers. Although pesticides are developed to function with reasonable certainty and minimal risk to human health and the environment, many studies have raised concerns about health risks from exposure of farmers (or other end-users of pesticides) and from non-occupational exposure of the population to residues found on food and drinking water. Several indicators have been used to assess the potential risk of pesticides to human health and the environment. However, their use indicated reduced certainty, suggesting the need for development of alternative indicators that should increase the accuracy and reliability of pesticide risk assessment and thus contribute to reduction of the possible adverse effects of pesticides on human health and the environment.

The development of new pesticides with novel modes of action and improved safety profiles and the implementation of alternative cropping systems that are less dependent on pesticides could minimize exposure to pesticides and the undesirable effects of exposure on human health. Moreover, the use of appropriate and well-maintained spraying equipment along with taking all the precautions required in all stages of pesticide handling could also reduce exposure to pesticides. The overall optimization of pesticide handling strictly according to the regulations and also considering the public concerns about pesticide residues in food and drinking water could contribute to reduction of the adverse effects of pesticides on human health and environment. All these may sound difficult, but seem to be a promising way for sufficient supply of safe food production within a viable agricultural production system.

## Figures and Tables

**Table 1.** Acute toxicity of pesticides according to WHO classification (adapted from [30]).

**Class**	**Classification**	**LD_50_ for the rat (mg/kg b.w.)**			
		**Oral**		**Dermal**	

		**Solids**	**Liquids**	**Solids**	**Liquids**
Ia	Extremely hazardous	<5	<20	<10	<40
Ib	Highly hazardous	5–50	20–200	10–100	40–400
II	Moderately hazardous	50–500	200–2,000	100–1,000	400–4,000
III	Slightly hazardous	>501	>2,001	>1,001	>4,001
U	Unlike to present acute hazard	>2,000	>3,000	–	–

**Table 2.** Acute toxicity of pesticides according to the EPA classification (adapted from [29]).

**Class**	**Signal words**	**Acute toxicity to rat**		
		**Oral LD_50_ (mg/kg)**	**Dermal LD_50_ (mg/kg)**	**Inhalation LC_50_ (mg/L)**
I	DANGER	<50	<200	<0.2
II	WARNING	50–500	200–2,000	0.2–2.0
III	CAUTION	500–5000	2,000–20,000	2.0–20
IV	CAUTION (optional)	>5,000	>20,000	>20

**Table 3.** Acute toxicity of pesticides (eye and skin effects) according to the EPA classification (adapted from [29]).

**Class**	**Signal words**	**Acute toxicity to rat**	
		**Eye effects**	**Skin effects**
I	DANGER	Corneal opacity not reversible within 7 days	Corrosive
II	WARNING	Irritation persisting for 7 days	Severe irritation at 72 hours
III	CAUTION	Irritation reversible within 7 days	Moderate irritation at 72 hours
IV	CAUTION (optional)	No irritation	Mild or slight irritation at 72 hours

**Table 4.** The three level tests to assess pesticide toxicity on non-target organisms (adapted from [48]).

**Species**	**Tier 1 Acute toxicity**	**Tier 2 Reproduction test**	**Tier 3 Field test**
Birds (bobwhite quail or mallard ducks)	*LD_50_* (8–14 days)		Fish life cycle study
Freshwater fish (rainbow trout or minnows)	*LC_50_* (96 h)	Effects on spawning	
Aquatic invertebrate (Daphnia, shrimp)	*LC_50_* (48 h)	Full life cycle	
Non-target invertebrate (honey bee)	*LD_50_* (48 h)	Effects of residues on foliage	Pollination field test
Non-target invertebrate (earthworms)	*LC_50_* (14 days)	Effects of residues on foliage	
Aquatic plants (algae)	*LC_50_* (96 h)	Plant vigour	
Other beneficial species	*LD_50_* (48 h)		
